# PRISMA statement and PROSPERO

**DOI:** 10.1590/S1677-5538.IBJU.2017.03.02

**Published:** 2017

**Authors:** Wanderley Marques Bernardo

**Affiliations:** Faculdade de Medicina da Universidade de São Paulo (FMUSP), Projeto Diretrizes - Associação Médica Brasileira (AMB), São Paulo, SP, Brasil

Independent of who made the first Review in health literature, it is possible to state that the development of the fundamental concepts on Systematic Review and Meta-analysis is attributable to the Cochrane initiative, that signalized the need to gather scientific evidence based on the opinion of medical specialists (1). It is necessary to periodically organize a synthesis, by specialty, of all relevant randomized clinical trials, which stimulated, in 1993, the creation of collaboration, which has been at the forefront of the methodology and in the rigorous systematic review.

Key elements of the model are transparency and reproducibility search methods, which include registration of the title, publication of the protocol and periodic updating in subsequent systematic reviews. At same time at the UK Cochrane Center in Oxford, it was initiated the development of statistical methods for data synthesis, which great influenced on the specifications of the software, especially in relation to meta-analysis (2). Throughout these years, the methodology has been actively improved, including the development of risk assessment instruments for bias in no randomized studies (1).

The items involved in the development of the systematic review, specifically for interventions based on randomized assays, also had their initial definition by the Cochrane Handbook (1994), inserted in its software (RevMan). But in the same way as systematic revisions, it has become a universal process, as well as initiatives have emerged to disseminate these fundamental elements, such as the PRISMA (Preferred Reporting Items for Systematic Reviews and Meta-Analyzes) (3), which has its origins in QUOROM (Quality of Reporting of Meta-Analyses) (4), which was a specific instrument for meta-analyzes of Randomized Clinical Trials (1996).

Some major phenomena justified the improvement, the democratization and the expansion of the checklist for systematic reviews, through the creation of PRISMA (2005): the knowledge of driving and publication of systematic reviews has expanded, the concepts of risk of bias were extended, including observational studies, and the synthesis of the evidence used by the authors has become increasingly focused on to address practical issues, making the eligibility criteria of the increasingly inclusive evidence. The PRISMA consists of a “checklist” of 27 items and a 4-stage flow diagram essential for the dissemination and publication, transparent and rigorous, of the methods and results of the systematic review (3).

In the same way, to broaden the global Systematic Reviews of quality, the Center for Reviews and Dissemination (CRD), at York University, which is part of the National Institute of Health Research (NIHR) in the UK, and produces systematic reviews and evaluations of health technology, has developed a record of systematic reviews, the international prospective register of systematic reviews (PROSPERO) (5). PROSPERO provides the first base to register systematic reviews in health, and through a broad consultation promotes best practice around the World, in order to reduce redundancy, and wasting time and resources.

So Int Braz J Urol is adopting the PRISMA checklist, including the PROSPERO’s register, within the rules needed to submitted any systematic review with or without meta-analysis. The authors can access the checklist and flow diagram at Prisma-Statement and the registration at Prospero electronic address, that need to be sent with the whole submission.

Certainly, with this initiative, the level of the Systematic Review and Meta-analysis available in the Int Braz J Urol will be with highest level, consistence and credibility.

Wanderley Marques Bernardo Faculdade de Medicina da Universidade de São Paulo (FMUSP), Projeto Diretrizes - Associação Médica Brasileira (AMB), São Paulo, SP, Brasil
